# Autonomous growth potential of leukemia blast cells is associated with poor prognosis in human acute leukemias

**DOI:** 10.1186/1756-8722-2-51

**Published:** 2009-12-29

**Authors:** Ying Yan, Eric A Wieman, Xiuqin Guan, Ann A Jakubowski, Peter G Steinherz, Richard J O'Reilly

**Affiliations:** 1 The Saint Luke's Cancer Institute, 4321 Washington, Suite 4000 Kansas City, Missouri 64111, USA; 2 School of Medicine, University Missouri-Kansas City, Holmes Road Kansas City, Missouri 64108, USA; 3 Bone Marrow Transplantation Service and the Department of Pediatrics, Memorial Sloan Kettering Cancer Center, New York, New York, USA

## Abstract

We have described a severe combined immunodeficiency (SCID) mouse model that permits the subcutaneous growth of primary human acute leukemia blast cells into a measurable subcutaneous nodule which may be followed by the development of disseminated disease. Utilizing the SCID mouse model, we examined the growth potential of leukemic blasts from 133 patients with acute leukemia, (67 acute lymphoblastic leukemia (ALL) and 66 acute myeloid leukemia (AML)) in the animals after subcutaneous inoculation without conditioning treatment. The blasts displayed three distinct growth patterns: "aggressive", "indolent", or "no tumor growth". Out of 133 leukemias, 45 (33.8%) displayed an aggressive growth pattern, 14 (10.5%) displayed an indolent growth pattern and 74 (55.6%) did not grow in SCID mice. The growth probability of leukemias from relapsed and/or refractory disease was nearly 3 fold higher than that from patients with newly diagnosed disease. Serial observations found that leukemic blasts from the same individual, which did not initiate tumor growth at initial presentation and/or at early relapse, may engraft and grow in the later stages of disease, suggesting that the ability of leukemia cells for engraftment and proliferation was gradually acquired following the process of leukemia progression. Nine autonomous growing leukemia cell lines were established in vitro. These displayed an aggressive proliferation pattern, suggesting a possible correlation between the capacity of human leukemia cells for autonomous proliferation in vitro and an aggressive growth potential in SCID mice. In addition, we demonstrated that patients whose leukemic blasts displayed an aggressive growth and dissemination pattern in SClD mice had a poor clinical outcome in patients with ALL as well as AML. Patients whose leukemic blasts grew indolently or whose leukemia cells failed to induce growth had a significantly longer DFS and more favorable clinical course.

## Introduction

Acute leukemia originates from transformed normal hematopoietic progenitor cells. The leukemogenic transformation may require multiple steps at the molecular and cellular level. During the leukemic transformation, the stem cells could gradually acquire the potential for spontaneous proliferation, and abnormal apoptosis.

The autonomous growth potential of the leukemia blasts may result from autocrine stimulation and this may predict the prognosis in some type of leukemias [1-5]. A number of studies have shown that blasts from some patients with acute leukemia displayed either a partially or a totally autonomous growth pattern in liquid culture medium or in clonogenic assays [6-8]. However, most of the reports were based on studies of a short-term (3 or 7 days) culture of the leukemia blasts in serum-free medium by a ^3^H-thymidine incorporation assay or based on a 7 days colony forming assay in methylcellulose medium to predict the leukemia blast proliferation activity [2,4,9,10]. Under these conditions, a substantial portion of blasts die due to apoptotic pressure from the in vitro cell culture soon after the culture starts. The possible contamination by lymphocytes and monocytes may also interfere with the determination of autonomous growth of the leukemic blast cells. Thus, these methods may at best represent the temporary in vitro growth ability of the blast cells, but they may not accurately reflect the individual, intrinsic, spontaneous proliferation and long-term proliferation potential of leukemic blasts.

Recent studies have demonstrated that growth of some human leukemia cells in the severe combined immunodeficiency (SCID) mouse model is analogous to their biological characteristics in patients. This method has allowed human leukemic cells which in prior studies failed to grow adaptively under in-vitro culture conditions to propagate in SCID mice. The consistent ability of SCID mice to propagate human leukemic cells thus provides a new vehicle for studying the biologic characteristics and proliferation potential of the various leukemia cells [11-15].

We have developed a SCID mouse model that permits the subcutaneous growth of primary human acute leukemia blast cells and cells from the myeloid blast crisis of chronic myelogenous leukemia [16,17]. The subcutaneous engraftment and growth of human leukemias in this model are associated with dissemination of leukemia blasts and reflect a pattern of growth similar to the usual clinical presentation. Furthermore, human acute leukemias in SCID mice displayed three distinct growth patterns: aggressive, indolent or no growth [16]. In this report we demonstrate that the proliferation ability of human leukemias in a SCID mouse model is associated with prognosis in acute human leukemias.

## Materials and methods

### Ptients

Patients with newly diagnosed or relapsed acute leukemias at Memorial Sloan-Kettering Cancer Center (MSKCC) were included in this study. One hundred thirty three patients were studied after providing informed consent. Their diseases were classified according to the French-American-British (FAB) classification, based on the morphology of cells as examined by light microscopy. The clinical and hematologic characteristics of the patients are summarized in Table 1. The mean age of the patients was 20 years (range, 3 to 76). Eighty six patients were male and 47 were female with a M/F ratio of 1.83/1. Out of 67 acute lymphoblastic leukemias (ALLs), 27(40%) were newly diagnosed, 38(57%) had recurrent disease, and 2 patients (3%) with T-ALL had primary refractory leukemia. In the patients with B-lineage-ALLs, 22/53 pre-B-ALLs were newly diagnosed and 6 of them demonstrated high risk features (WBC >50,000/μl and/or age >10 years); 31 pre-B-ALLs and 3 B-cell lymphoma/leukemias (B-ALL L3) had relapsed disease. In T-ALLs, 5/11 patients were newly diagnosed and 3 of them demonstrated high risk features; 6/11 T-ALLs had relapsed and/or refractory disease. The mean age of patients with ALL was 12 years (range, 1 to 54) and the M/F ratio was 1.79/1. Out of 66 patients with acute myeloid leukemia (AML), 35(53%) were newly diagnosed, including 2 patients (AML M2 and M4) who had a history of myelodysplasia (MDS) and 3 patients (AML M1, M4 and M5) diagnosed as secondary leukemia after treatment for osteogenic sarcoma, desmoplastic small round cell tumor, and neuroblastoma, respectively; 29(44%) patients had relapsed disease and 2(3%) had primary refractory leukemia. The mean age of the patients with AML was 27 years (range, 1 to 75) and the M/F ratio was 2/1.

**Table 1:** Characteristics of 133 patients with acute leukemias

Characteristic	Patients No. (%)
Immunophenotype and FAB Category of ALL	**67 (50.4)**
B-linage-ALL	**56 (42.1)**
Pre-B-ALL (L1 and L2)	**53 (39.8**)
Lymphoma/leukemia B-ALL (L3)	**3 (2.3)**
T-ALL (L1 and L2)	**11 (8.3)**
FAB Category of AML	**66 (49.6)**
M1 myeloblastic without maturation	**19 (14.3)**
M2 myeloblastic with maturation	**13 (9.8)**
M3 promyelocytic	**13 (9.8)**
M4 myelomonocytic	**11 (8.3)**
M5 monoblastic, monocytic	**5 (3.8)**
M7 megakaryoblastic	**3 (2.3)**
Unclassified	**2 (1.5)**

All patients received induction chemotherapy and were assessed for signs of a complete remission (CR) after one or two courses of therapy. Patients achieving CR were treated with at least two cycles of consolidation chemotherapy (AML) and risk group adjusted maintenance chemotherapy (ALL). Fifteen patients with ALL underwent bone marrow transplantation (BMT) (13 allogeneic and 2 autologous), and 26 patients with AML received BMT (23 allogeneic and 3 autologous). A complete remission was defined by a marrow with less than 5% blasts, normal hematopoiesis with normal peripheral blood counts, and disappearance of extramedullary leukemic cell infiltration.

Immunophenotype of the blasts was determined by flow cytometry [16]. Standard cytogenetic techniques, including cell preparations, cultures and banding techniques were used for examining the karyotype of patient-derived leukemic blasts as well as for the cells recovered from leukemic nodules grown in SCID mice.

### Inoculation of human leukemic cells into SCID mice

Samples were obtained, with informed consent, during routine diagnostic blood studies or bone marrow (BM) aspirates from patients with newly diagnosed or relapsed acute leukemia. Blast-enriched mononuclear cells were isolated by Ficoll Hypaque density gradient separation and washed in RPMI 1640 medium. After separation, most of the leukemic cells were freshly inoculated into SCID mice. In some patients leukemic cells were cryopreserved in liquid nitrogen prior to their injection into the animals. SCID (CB17-scid/scid) mice were purchased from Taconic Farms and maintained in microisolater cages in the animal laboratory under sterile conditions with a specific pathogen-free environment without the use of any antibiotics. Female SCID mice between 6-8 weeks of age were used. The method of inoculation of leukemia cells into SCID mice has been described [16]. Viability of the cells was determined by trypan blue staining. 1-2 × 10^7 ^viable leukemic cells were injected subcutaneously into the right flank of the SCID mouse. The number of SCID mice inoculated and the cell dose per mouse were dependent on the number of leukemic cells available from patient samples. Leukemic cell growth was assessed by weekly measurements of dimensions of the subcutaneous nodules.

For secondary passage of leukemia cells, the cells were harvested from tumor tissue removed from SCID mice and inoculated into fresh animals with a similar cell dose and method used as for the first passage. To determine the dissemination of human leukemia cells into distal organs of the animals, tissue sections from sacrificed SCID mice were prepared and stained according to standard techniques for histopathology assay. Fluorescence in Situ Hybridization(FISH) was performed as previously described [16].

### Long-term in vitro culture of leukemic cells

Blast-enriched mononuclear cells isolated by Ficoll Hypaque from patient-derived samples or mononuclear cells recovered from leukemic nodules were washed and plated into α-MEM medium containing 10% Fetal calf serum, penicillin (100 U/ml), streptomycin (100 μg/ml) and L-glutamine 2 mmol/L (Sigma) at a density of 5 × 10^5 ^cells/ml in 37°C, 10% CO^2 ^and fed one or twice a week.

### Statistical analyses

For statistical analysis, overall (OS) survival of patient was determined from the time of initial diagnosis until death from any cause or to last follow-up. Disease-free survival (DFS) was measured from the time of the first complete remission after induction therapy until the time of the first relapse after complete remission, or to the date of last follow-up, if none of the preceding events had occurred. Curves of OS and DFS of the patients with the various leukemia growth patterns in SCID mice were constructed using the method of Kaplan and Meier, statistical significance was determined with the Log-rank test and Wilcoxon test.

## Results

### Engraftment and growth of human acute leukemias in SCID mice

Leukemic blasts derived from each of the 133 leukemia patients were subcutaneously inoculated into SCID mice. In several patients, specimens had been serially collected at different times during the course of their disease, and inoculated into the animals both at diagnosis and at relapse. However, the calculation of engraftment and growth rate was based on the initially obtained sample. Out of 133 patients, leukemia blast samples from 59 (44.4%) engrafted and adoptively grew in SCID mice. In relation to clinical status, the probabilities of engraftment and growth of human acute leukemias in SCID mice were 14/62 (22.6%) for the newly diagnosed leukemias and 45/71 (63.4%) for the relapsed and/or refractory disease cases, respectively.

The engraftment and growth pattern of leukemic blasts from ALL and AML were listed in Table 2. Thirty of 67 (44.8%) patients derived ALL cells were able to grow in SCID mice. The growth rates were similar in B-lineage-ALL (25/56; 44.6%) and T-ALL (5/11; 45.5%). Five (18.5%) samples from 27 newly diagnosed ALL were able to grow in the SCID mice. In contrast, 25 (62.5%) samples from 40 relapsed or refractory ALL patients were able to induce subcutaneous tumor growth. Similarly, 9 out of 35 (25.7%) samples derived from newly diagnosed AML patients were able to grow in SCID mice. In contrast, 20/31(64.5%) relapsed AML samples were able to induce subcutaneous tumor growth and dissemination.

**Table 2:** Probability of engraftment and growth of human ALL and AML blasts in SCID mice by subcutaneous inoculation

Leukemias	**No**.	Engraftment & Growth (%)	p-value
**ALL**	67	30 (44.8%)	
B-lineage-ALL	56	25 (44.6%)	
new	22	5 (22.7%)	
rel/ref	34	20 (58.8%)	p < 0.05
T-ALL	11	5 (45.5%)	
new	5	0 (0%)	
rel/ref	6	5 (83.3%)	p < 0.01
**AML**	66	29 (43.9%)	
new	35	9 (25.7%)	
rel/ref	31	20 (64.5%)	p < 0.01

new = newly diagnosis; rel/ref = relapse/refractory

In general, a similar anatomic-pathologic picture was observed in most of the mice with leukemic engraftment. SCID mice bearing fast growing human leukemia tumors might develop axillary lymph node enlargement, hepatosplenomegaly, mediastinal lymphadenopathy or mesenteric lymphadenopathy. Histopathologic, FISH, and FACS analysis showed marked infiltration by human leukemic cells in bone marrow, peripheral blood, spleen, liver and other organs. In contrast, in animals with no leukemia engraftment, there was no appreciable splenic or liver enlargement or other clinical signs that would indicate leukemia development, and there was no evidence of leukemic cell infiltration detectable by FISH, FACS or histopathological analysis of the tissues.

### In vitro growth potential of the leukemia cells

**Figure 1:**
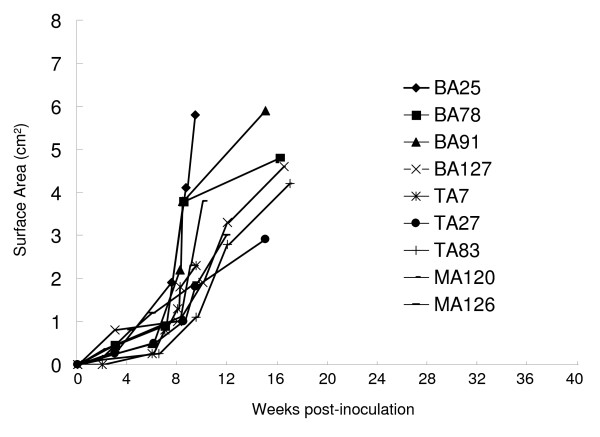
**Aggressive growth pattern of 9 acute leukemia cell lines in SCID mice**.

We cultured cells from 102 patient-derived samples as well as 45 samples recovered from leukemic nodules in vitro without cytokine supplementation. Nine leukemic cell lines developed from different patients were autonomously grew up and established in vitro. The characteristics of the patients whose leukemia blasts developed into cell lines are listed in Table 3. These 9 patients died soon after collection and inoculation of the specimens from which the cell lines were established. Eight cell lines were derived from relapsed patients. One was from a patient with secondary AML that arose after treatment for desmoplastic small cell tumor. The blasts, which were the source of these cell lines, displayed an aggressive growth pattern in all 9 cases (Figure 1), suggesting a possible correlation between autonomous in vitro proliferation and an aggressive growth pattern in vivo.

**Table 3:** Characteristics of the leukemia cell lines established in long-term in vitro cultures

Leukemia cell lines	Classifications	Clinical Status	Survival* (months)	Kayotypes
BA25**	B-ALL (L3)	Relapse-1/refractory	1.3	45, xy, t(2;8)(p12;q24),-4,+7,-10, idemx2; 44-47, t(2:8)(p23, q24)
BA78**	B-ALL (L3)	Relapse-1/refractory	0.4	45-47, XY, t(8;14)(q24, q32),-16 +1
BA91	B-ALL (L3)	relapse-1	4.5	45-46, XY, t(8;14)(q24, q32), -9, t(11;18)(q13;p11), +14, -16, +mar1, +mar2, +mar3
BA127	Pre-B-ALL (L1)	relapse-2	0.8	nd
TA7**	T-ALL (L1)	relapse-1	6.0	46, XY
TA27	T-ALL (L1)	relapse-1/refractory	3.0	46, XY
TA83	T-ALL (L1)	relapse-2/refractory	1.5	46, XY, del(9)(p21), -7, t(11;14)(p13;q11.2) -7
MA120**	AML (M2)	relapse-2/refractory	2.5	46, XY, del (16)(q13q22), dup(1) (q44q25)
MA126	AML (M4)	secondary leukemia	1.1	46, XY, t(9;11)(p21, q23)

*Survival of patients after leukemia specimens had be taken; nd: not done.

** Cell lines established from leukemic nodules in SCID mice. The primary specimen did not grow.

### Growth potential of leukemia blasts derived from the same patient is different during different clinical stages

**Figure 2:**
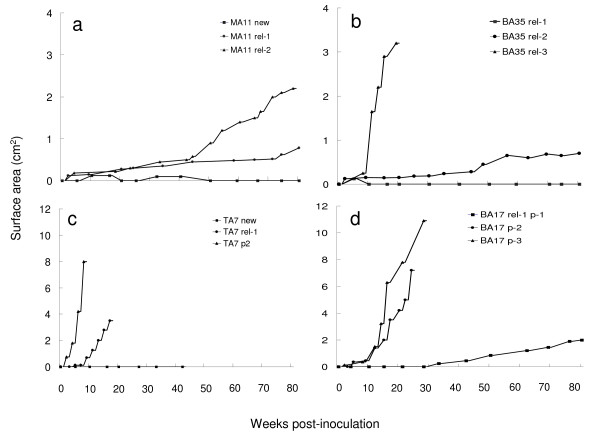
**Engraftment and growth patterns of leukemia blasts derived from individual patients with different clinical status in SCID mice**. a. Leukemia blasts obtained from MA11 at initial diagnosis did not engraft and growth in SCID mice. The cells collected from her first relapse (rel-1) and second relapse (rel-2) was grown in the mice in an indolent manner, respectively. The patient died of leukemia 2 months later after the injection of rel-2 cells. b. BA35 was a patient with pre-B-ALL. Leukemia sample from initial diagnosis was not studied. The blasts of rel-1 were not able to engraft and grow. Cells from rel-2 and rel-3/ref displayed an indolent and aggressive growth pattern, respectively. The patient died of leukemia 3 months after refractory disease development. c. TA7 was from a newly diagnosed patient with T-ALL. Leukemia cells from initial diagnosis did not grow. Cells from first relapse had an aggressive growth pattern. Leukemic cells recovered from subcutaneous tumor, were able to initiate a more rapid proliferation than the cells in first passage. d. BA17 was a pre-B-ALL who relapsed after a 40 months complete remission and died of leukemia progression 3 months after relapse. His leukemia cells from rel-1 displayed an indolent growth. However, the adoptive growth in second and third passages displayed an aggressive growth pattern.

To investigate whether or not the growth potential of leukemia blasts at different clinical stages of the disease is different, leukemia specimens were serially collected from the same patient at initial presentation and at relapse. Leukemia blasts derived from a newly diagnosed AML patient (MA11 M4) were not able to engraft and grow in SCID mice, however, the cells collected at the time of the first and second relapse (rel-1 and rel-2) engrafted and grew in mice in an indolent manner. The cells from rel-1 grew very slowly. Tumor size did not achieve 1 cm^2 ^until 80 weeks after inoculation. In contrast, the tumor size was 2.2 cm^2 ^by week 78 after inoculation with cells from rel-2 (Figure 2a.). In the case of a pre-B-ALL (BA35) patient, with leukemia cells from rel-1 were not able to engraft and grow. The cells collected during rel-2 and rel-3 displayed an indolent and aggressive growth pattern, respectively. The tumor originated from relapse 2 reached a surface area of 0.8 cm^2 ^at week 80 after inoculation. And cells from relapse 3 grew into a tumor with a surface area of 3.1 cm^2^at 18 weeks after relapse-3 (Figure 2b.). The patient died at 3 months after her third relapse. A similar observation was seen in a patient with T-ALL (TA7) (Figure 2c.), with no growth at initial presentation. Cells at relapse demonstrated an aggressive growth pattern. This tumor achieved a surface area of 3.5 cm^2 ^within 15 weeks. Survival of this T-ALL patient was less than 6 months after his relapse.

In vivo propagation potential of human leukemic cells in SCID mice was studied in cells originally derived from TA7 and a pre-B-ALL (BA17) (Figure 2c and 2d.). Cells harvested from subcutaneous tumor nodule were inoculated subcutaneously into secondary SCID mice with the same cell number used in the original passage. The growth of each subsequent passage of the leukemia cells was significantly more rapid than during the first passage. The TA7 leukemic cells grew to a tumor size 8 cm^2 ^in 7 weeks during secondary passage while they were not measurable at this time during the primary passage. In patient BA17, leukemia blasts derived from rel-1 induced an indolent growth pattern and only achieved a tumor size of 2 cm^2 ^by 80 weeks. However, the cells in the second and third passages displayed an aggressive growth by 23 weeks, the tumor sizes were 5 and 8 cm^2^, respectively.

### Relationship between growth patterns of leukemic cells and clinical stages

In a previous study [16], we found that there were 3 distinguishable growth patterns for human acute leukemias in SCID mice: aggressive growth, indolent growth and no growth. Similar growth and dissemination patterns of the leukemias were demonstrated in the current study. Out of 133 leukemias, 45 (33.8%) present an aggressive growth pattern, 14 (10.5%) had an indolent growth pattern and 74 (55.6%) did not grow in SCID mice. The mean time for the tumors to reach 1.0 cm^2 ^and 2.0 cm^2 ^surface area was 11.4 ± 3.5 and 15.2 ± 5.2 weeks in those with an aggressive growth pattern (Table 4). In contrast, the mean time for the tumors to reach 1.0 cm^2 ^and 2.0 cm^2 ^were 35.9 ± 13.1 and 43.9 ± 14.1 weeks, respectively, for those with an indolent growth pattern.

**Table 4:** Mean Time of Leukemic Tumor Achieved 1.0 cm^2^and 2.0 cm^2 ^of Surface Areas

Leukemia category and growth patterns	1.0 cm^2 ^(weeks)	2.0 cm^2 ^(weeks)
Leukemia (overall)		
Aggressive n = 45	11.4 ± 3.5 (n = 45)	15.2 ± 5.2 (n = 39)
Indolent n = 14	35.9 ± 13.1 (n = 14)	43.9 ± 14.1 (n = 12)
AMLs		
Aggressive n = 22	12.1 ± 4.2 (n = 22)	17.4 ± 6.7 (n = 19)
Indolent n = 7	31.1 ± 6.2 (n = 7)	40.1 ± 7.8 (n = 6)
B-linage-ALLs		
Aggressive n = 18	11.9 ± 4.3 (n = 18)	14.8 ± 5.4 (n = 16)
Indolent n = 7	43.1 ± 16.1 (n = 7)	50.3 ± 15.7 (n = 6)
T-ALLs		
Aggressive n = 5	9.5 ± 2.9 (n = 5)	11.3 ± 2.9 (n = 5)

The tumor growth patterns for the different types of leukemia are listed in Table 4. Eighteen out of 56 (32%) B-lineage-ALLs displayed an aggressive growth pattern and 7/56 (13%) had an indolent growth pattern. These correlated with the clinical status; 15/18 (83%) B-lineage-ALLs that grew aggressively and 4/7(57%) that had an indolent pattern, were derived from patients with relapsed and/or refractory disease, respectively. All the samples from 5 T-ALLs (4 relapsed and 1 refractory disease) that engrafted in SCID mice, displayed an aggressive growth pattern. 17 of 22 (77%) AML leukemia samples with an aggressive growth pattern were derived from patients with relapsed/refractory disease.

### No growth and indolent growth patterns in SCID mice suggest a more favorable clinical outcome for ALL and AML patients

We followed 66 ALL patients. They are separated into three groups according to the growth patterns of their leukemic cells in SCID mice. 23 patients' leukemic blasts (18 B-lineage-ALL and 5 T-ALL) presented an aggressive growth pattern; 7 patients with pre-B-ALL displayed an indolent growth and 36 (30 pre-B-ALL and 6 T-ALL) had "no growth".

**Figure 3:**
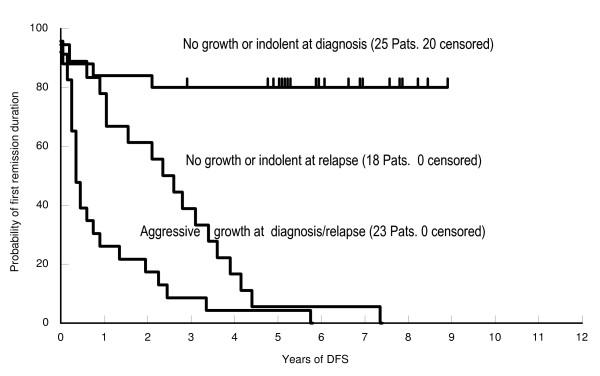
**Probability of first remission duration of ALL patients in relation to the growth pattern of their leukemic blasts in SCID mice**. Patients whose leukemic cells grew aggressively (newly diagnosed or relapsed, n = 23) had a poor clinical outcome. Patients whose leukemic blasts displayed either no tumor growth or an indolent growth pattern at newly diagnosis (n = 25) had a favorable clinical outcome. Patients whose leukemic blasts displayed no tumor growth or an indolent pattern at relapse (n = 18) also had a relatively favorable clinical outcome.

First remission duration of patients with ALL in these three groups is demonstrated in Figure 3. Patients, whose leukemic cells exhibited an aggressive growth pattern, had a very poor clinical course no matter whether the cells were obtained at diagnosis or at relapse. The median DFS was 0.375 years (0-5.9 years) and median OS was 1.42 years (0.2-6.9 years). Twenty two Patients died of progressive disease, with the exception of one pre-B-ALL patient, who survived 6.9+ years in his third remission.

In contrast to the "aggressive group", a more favorable clinical outcome was found in newly diagnosed patients with "no growth" and "indolent" growth. The mean DFS and OS was 5.3 years (p < 0.001) and 5.5 years (p < 0.001), respectively. Twenty out of 25 patients in this group were relapse free and only 5 patients died of leukemia progression. Patients with relapsed disease, who did not have aggressive growth, had an intermediate outcome with a 2.6 year (p = 0.004) and 4.3 year (p = 0.002) mean DFS and OS, respectively. However, 11/18 patients died of leukemia progression. Seven patients maintained their remission (6 in CR2 and 1 in CR3).

**Figure 4:**
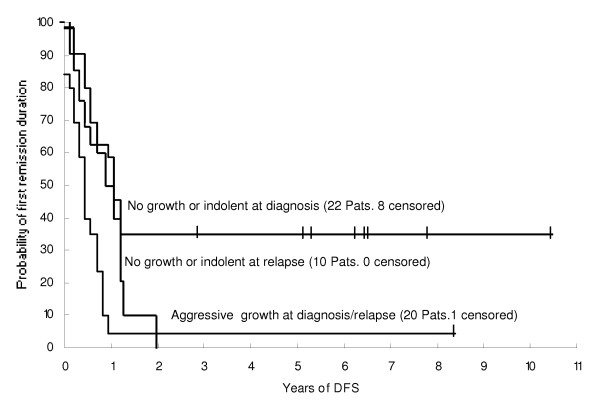
**Probability of first remission duration of AML patients in relation to the growth patterns of their leukemic blasts in SCID mice**. Patients (newly diagnosed or relapsed, n = 20) whose leukemic cells grew aggressively had a poor clinical outcome. In contrast, patients whose leukemic blasts displayed either no tumor growth or an indolent growth at initial presentation (n = 22) had a favorable clinical outcome. Patients whose leukemic blasts displayed no tumor growth or an indolent pattern at relapse (n = 10) also had a poor out come.

Of the 66 AML patients studied, one (M2) had incomplete clinical data available and the result of the 13 acute promyelocytic leukemias (APL) will be reported separately below. The 52 remaining patients were separated into 3 groups according to the growth pattern of their leukemic cells as above. Twenty relapsed (n = 13) or newly diagnosed (n = 7), had an aggressive growth pattern. These patients had a poor clinical outcome, with a median DFS of 0.32 (0-8.3) years (Figure 4.). Only one patient is still in CR, 8.3 years after an allogeneic BMT. The other 19 patients relapsed within a year. Eighteen patients had died of leukemia progression. The OS in these patients was 0.68 (0.2-9.8) years.

A more favorable clinical outcome was found in the patients with "no growth" or "indolent" growth. Twenty-two of these patients were studied at diagnosis. Eight had maintained at CR1 and 14 (63.6%) died of leukemia progression. The median DFS was 1.0 (0.1-10.5) years, significantly longer than that of the aggressive growth group (p = 0.018). The median OS was 1.2 (0.1-10.7) years, longer than that of the aggressive growth group, although the difference was statistically insignificant (p = 0.36). Only 7 newly diagnosed patients had an aggressive growth pattern. Six (85.7%) patients died within one year except one is still alive, with no evidence of disease 8 years after achieving remission. Their median DFS and OS were 0.23 (0.05 - 8.3) and 0.46 (0.1-8.6) years, respectively, shorter than that of the "no growth" or "indolent" growth group. However, there were too few cases, which had an aggressive pattern at diagnosis, to archive a statistical significance. Therefore, a future study including a larger number of patients would be necessary to further confirm a poor prognosis in the patients who had an aggressive growth pattern at diagnosis.

Thirteen APLs had been included in this study, from whom 5 patients derived leukemia sample were able to engraft and grow in SCID mice. Out of 6 newly diagnosed APLs, 1 patient whose leukemia cells grew in the animals, displayed an indolent growth pattern. In 7 APLs with relapse disease, leukemia blasts from 4 patients displayed either aggressive growth (n = 2) or indolent growth pattern (n = 2), respectively. The mean times of their leukemic tumor size arrived to 1.0 cm^2 ^and 2.0 cm^2 ^surface areas were 15.9 ± 3.6 and 21.4 ± 5.8 weeks for the 2 patients with aggressive pattern, and 33.2 ± 8.8 and 41.7 ± 14.4 weeks for the 3 APL with indolent growth pattern, respectively. The probability of engraft and growth of APL leukemia 5/13 (38%) seems compatible with the general AML group (Table 2); however, the clinical outcome was obviously more favorable for the APL patients. The median DFS and OVS were 4.4 and 8.6 years, respectively, for the entire group of the APL patients.

## Discussion

A number of factors have been shown to have prognostic values in human acute leukemias. For example: the biological characteristics of the leukemic cells such as morphology phenotype, specific karyotypic abnormalities and response to therapy have prognostic values in the leukemias. Studies have demonstrated that the in vitro autonomous proliferation potential of patient derived leukemia blasts can influence ALL as well as AML prognosis [1-4]. Lowenberg et al. reported an in vitro study which measured the uptake of tritiated thymidine by leukemic cells in serum-free and cytokine-free cultures as a means of determining the rate of spontaneous proliferation in 114 newly diagnosed AML patients. In that study, leukemia blasts displayed either a low, intermediate, or high proliferation pattern, and patients with high rates of proliferation activity had a poor prognosis [1]. The capacity for autonomous proliferation of leukemia blasts is associated with the degree of aggressiveness of acute leukemia [1,4].

In the present study, we examined the ability of patient-derived acute leukemia cells to grow and disseminate in SCID mice by subcutaneous inoculation without conditioning treatment or administration of growth promoting cytokines. Leukemic cells from 59 of 133 (44.4%) patients were successfully engrafted into mice. In our SCID mouse model, patient-derived leukemia blasts also displayed three distinct growth patterns: either aggressive, indolent or no growth. We correlated the growth patterns of blasts to the status of patients during follow up studies. We observed that the aggressive growth pattern of leukemic cells derived from ALL as well as AML patients is correlated with a poorer clinical outcome. No growth and indolent growth are correlated with a more favorable outcome. Our observation is in agreement with the previous studies.

In our study, 26% of samples from newly diagnosed AML could engraftment and growth in the animals. In recent publications, Sanchez et al demonstrated a higher engraftment rate (37% with ≥ 10% leukemia cells infiltrating marrow, plus 14% ≥ 1% infiltrating leukemia cells) after intravenous injection of samples from patients presenting with AML into a IL2R NOD/SCID mice model [15]. The IL2R NOD/SCID mice, which have been depleting T, B as well as NK cells function. This results a higher immune competent potential. They also treated the animals with sublethal irradiation (250 cGy of total body irradiation) 24 hours before IV injection of leukemic cells. All these factors may influence and increase the AML sample engraftment and growth rate in the animals. In contract, we used CB17-scid/scid mice (homozygous for the severe combined immune deficiency characterized by an absence of functional T and B cells, but maintaining functional NK cells) without any conditioning treatment before inoculation. This might lead to a lower engraftment and growth rate. Therefore, introduction of the IL2R NOD/SCID mice as well as the immunosuppressive conditioning treatment before inoculation may improve leukemia growth rate in this subcutaneous SCID mouse model in future studies.

In correlation to clinical status, the probability of engraftment and growth of leukemia blasts collected during relapse reaches 63%, which is nearly 3 times higher than the growth potential of blasts collected upon initial diagnosis (23%). The ability of patient-derived blasts to engraft and grow in SCID mice was also enhanced as the same patient progressed in his clinical stages. We also observed the engraftment and growth potential of leukemia cells is enhanced accompanying each subsequent relapse (Figure 2). This observation is in agreement with our previous study on chronic myelogenous leukemia (CML) in the same SCID mice system. The leukemia cells from chronic and accelerated phase had very little or no permanent engraftment and growth potential, however, cells from blast crisis grew and disseminated [17]. All these facts suggested that the ability of leukemia blasts engraftment and proliferation was gradually acquired along the progression of leukemia in most patients.

ALL patients whose leukemic cells had a low growth potential enjoyed a better OS and DFS as compared to patients whose cells had high growth potential in each group. This observation suggested that patients whose leukemia cells maintained lower proliferation characteristics responded to treatment better.

Similar to those with ALL, patients with newly diagnosed AML whose leukemia cells had no growth or indolent growth pattern had a better DFS than the patients with an aggressive pattern, suggesting that an aggressive growth pattern can serve as an index for worse prognosis of human AML as well. However, we found that relapsed AML patients whose cells had indolent growth did not demonstrate a significantly better OS than the relapsed patients with an aggressive growth pattern. This might suggest that factors other than the aggressive growth pattern of human leukemic cells might be of prognostic value.

We established 9 in vitro leukemia cell lines out of 147 leukemia specimens. The in vitro cell lines may represent leukemia cells with maximal autonomous growth potential. All the patients from whom the cell lines derived were in their later stages of the disease when the samples were obtained. All the leukemia cell lines displayed an aggressive growth pattern in SCID mice, suggesting that the in vitro growth potential of the leukemia blasts is correlated and consistent with their in vivo growth potential.

In this study, leukemia cells from 38.5% APL patients were able to induce engraftment and growth in SCID mice, with no significant difference to other AMLs in SCID mice. However, the clinical outcome was obviously favorable for the APLs, in comparison with other leukemias, suggesting an effect therapeutic strategy could be important to the leukemia patients, even with an aggressive disease. Improved therapy for APL, which changes the prognosis of APL from a fatal leukemia to a highly curable disease, is one of the major achievements of leukemia research, reflecting the accomplishment from the combination of progress in both laboratory science and well-designed clinical trials [18-23]. Due to the limited number of APLs in this study, it would be necessary to study more patients to clarify and confirm if and why the APL blasts grow in SCID mice despite a favorable clinical outcome. In addition, considering the heterogeneity of the AML patient samples in this study, a larger number of patients with the specific AML subtypes need to be studied to determine any correlations between the clinical outcome and the incidence of the different patterns of leukemic growth in SCID mice.

In conclusion, the in vivo growth characteristics of the leukemic blasts can be considered as an important prognostic factor in ALL and in AML. The ability of leukemia blasts to engraft and proliferate is gradually acquired following leukemia progression in most patients. The growth characteristics of the leukemic blasts should be considered in the assignment of patients to different therapeutic options. In addition, a prospective study can be included in the future to evaluate the molecular mechanism that contributes to the different growth characteristics of the leukemic blasts.

## Competing interests

The authors declare that they have no competing interests.

## Authors' contributions

YY carried out the animal experiments and participated in the design of the study and research coordination. EW carried out research data and statistics analysis and drafted the manuscript. XG participated in animal experiments. AJ participated in patient specimens and clinical data collection. PS carried out clinical research, coordinated patient specimen and clinical data collection and drafted the manuscript. RO conceived of the design of the study and coordination. All authors read and approved the final manuscript.
